# Integrating Classical Preprocessing into an Optical Encryption Scheme

**DOI:** 10.3390/e21090872

**Published:** 2019-09-07

**Authors:** Hai Pham, Rainer Steinwandt, Adriana Suárez Corona

**Affiliations:** 1 Department of Mathematical Sciences, Florida Atlantic University, Boca Raton, FL 33431, USA (H.P.) (R.S.); 2 West Campus Mathematics Division, Valencia College , Orlando, FL 32811, USA; 3 Department of Mathematical Sciences, Universidad de León, 24071 León, Spain

**Keywords:** symmetric encryption, all-or-nothing transform, optical channel, provable security

## Abstract

Traditionally, cryptographic protocols rely on mathematical assumptions and results to establish security guarantees. Quantum cryptography has demonstrated how physical properties of a communication channel can be leveraged in the design of cryptographic protocols, too. Our starting point is the AlphaEta protocol, which was designed to exploit properties of coherent states of light to transmit data securely over an optical channel. AlphaEta aims to draw security from the uncertainty of any measurement of the transmitted coherent states due to intrinsic quantum noise. We present a technique to combine AlphaEta with classical preprocessing, taking into account error-correction for the optical channel. This enables us to establish strong provable security guarantees. In addition, the type of hybrid encryption we suggest, enables trade-offs between invoking a(n inexpensive) classical communication channel and a (more complex to implement) optical channel, without jeopardizing security. Our design can easily incorporate fast state-of-the-art authenticated encryption, but in this case the security analysis requires heuristic reasoning.

## 1. Introduction

The fast development of telecommunications and the increase of (potentially sensitive) data stored and exchanged by companies or individuals through public networks has made cryptography particularly important to guarantee the privacy, integrity and authenticity of users. The current approach to protect data involves the use of combinations of secret key [1] and public-key solutions [2], which base their security on empirical evidence or on the difficulty of solving certain mathematical problems respectively. A different approach has been explored since the 1980s, after Bennet and Brassard [3] proposed their seminal quantum key distribution protocol. Today research in quantum cryptography goes well beyond quantum key distribution [4,5].

Quantum-based cryptographic schemes have the conceptual appeal that security guarantees can potentially be argued based on fundamental laws of physics. However, popular quantum protocols, based on [3,6], commonly rely on single photon sources, which can be challenging to implement. As a result, in recent years, the idea of using mesoscopic coherent states has gathered interest, and the AlphaEta protocol is a prominent example of such a design (see, e.g., [7,8,9,10]). Its security has been the topic of several papers, including [9,11,12,13,14]. AlphaEta is also cited in Lloyd’s work on quantum enigma machines [15]; Lloyd notes the open problem *to construct a provably secure quantum enigma machine using linear optics and coherent states.* As an alternative to AlphaEta, other optical cryptographic solutions were considered, including the use of double random phase encoding (DRPE) [16,17,18]. It deserves noting that not only has academia been focusing on quantum technologies, but several industries have started to commercialize quantum cryptographic tools [5], specifically for quantum key distribution and quantum random number generation.

Capturing security guarantees that rely on physical assumptions with common security models for encryption poses somewhat of a challenge, especially when trying to integrate computationally secure primitives as well. Barbosa and van de Graaf rightfully point out that protocols based on quantum optical noise appear to be a wonderful source of research questions [10], though. It is tempting to harvest both the strength of existing (computationally secure) efficient cryptographic constructions and the features offered by an AlphaEta-type protocol, where an eavesdropper faces an additional, physical, hurdle.

### Our Contribution

A protocol is proposed which takes advantage of the physical security guarantee offered from the AlphaEta setup and builds on this using classical constructions. In Section 2, we review the AlphaEta protocol and some definitions and results about *all-or-nothing transforms* (AONTs), a tool that has already proved to be useful in Li et al.’s work [19]. To be able to work conveniently with individual bits when discussing security, we introduce the notion of a *restricted* AONT and present a way of constructing these type of transformations. We propose a security model for encryption schemes using “hybrid ciphertexts”, invoking both an optical channel (like AlphaEta does) and a classical communication channel. We present an (efficient) construction, building on AlphaEta, offering security in our model.

The security guarantee we establish is information-theoretic: we leverage the optical channel so that (physical) guarantees should prevent the adversary from learning any bit of the payload. Standalone, this may not be satisfying for applications yet, but we can integrate classical (high-speed) authenticated encryption. Then, with a heuristic argument, we create a situation where an adversary is unlikely to even intercept the correct *ciphertext*, offering a conceptually interesting additional layer of security: in traditional attack models, knowledge of the ciphertext is commonly considered as granted. It seems fair to say that we offer the first formalized proposal dealing with such hybrid ciphertexts, provably establishing the aforementioned advantage. It adds the physical security guarantee, but is easier/more flexible to implement since not the whole ciphertext needs to be transmitted through the optical channel.

## 2. Background and Tools

A protocol we will make use of is AlphaEta, and we briefly review the essential pieces, following mainly [8].

### 2.1. The AlphaEta Protocol

Ciphertexts in AlphaEta are a sequence of light pulses, where each pulse consists of many photons. They are represented using coherent states, and throughout, we use the following notation:

∘ 〈n〉: average number of photons per pulse
∘ β: number of bases used
∘ *s*: number of pulses sent in one round of the protocol

The two communicating parties are assumed to share a uniformly random $\left(b_{1},\cdots ,b_{s}\right)\in {\left\{0,\cdots ,\beta -1\right\}}^{s}$ that is unknown to the adversary and determines the bases used. Given a plaintext $a=\left(a_{1},\cdots ,a_{s}\right)\in {\left\{0,1\right\}}^{s}$, the sender will transmit each bit $a_{i}$ in phase angle

(1) $$\varphi_{a_{i},b_{i}}=\left(\frac{b_{i}}{\beta }+a_{i}\right)\cdot \pi$$

to the receiver using the optical channel. Knowing the bases $b_{i}$, the receiver measures $\varphi_{a_{i},b_{i}}$ and maps it to the nearest phase angle to determine the original plaintext bit $a_{i}$.

The number of photons in each pulse follows a Poisson distribution with parameter 〈n〉. This  statistical fluctuation is the quantum noise, which is intrinsic and cannot be avoided. This 〈n〉 is also related to the phase angle φ used to modulate light pulses. Hence, there are fluctuations in the phase angle φ as well. The security of a protocol using coherent states is based on the difficulty of determining the right phase angle if the basis is not known. An eavesdropper, not knowing the basis, faces an intrinsic error that can be bounded from below by a value that can be made close to 1/2. Whereas the intended recipient, knowing the shared basis in which to measure, can recover any plaintext bit almost perfectly (bit error rates are below $10^{-9}$).

In [8], it is shown that if a plaintext bit is chosen uniformly at random, which is typical for a key transport application, then the minimum probability of error $P_{e}$ that an eavesdropper can achieve in the bit determination can be arbitrarily close to 1/2 by choosing the appropriate parameters β and 〈n〉 (see ([8] Figure 3)). Therefore, the entropy about a bit of the plaintext given the measurement is reduced by only a small quantity ϵ and the mutual information of both random variables approaches that value ϵ (see ([8] Figure 4)).

In the next section, we recall a theoretical tool which will enable us to split a payload between two different types of channels—an optical one and a classical one—without jeopardizing security.

### 2.2. All-or-Nothing Transforms

Let ℓ,s be positive integers such that 1≤ℓ≤s and *X* be a finite set with |X|=v. We say that $\phi :X^{s}⟶X^{s}$ is an *(ℓ,s,v)-all-or-nothing transform* (AONT) provided that all of the following holds:

1. ϕ is a bijection.
2. If any s−ℓ of the *s* output values $y_{1},\cdots ,y_{s}$ are fixed, then any *ℓ* of the input values $x_{i}$ (1≤i≤s) are completely undetermined, in an information-theoretic sense.

Following [20,21], throughout we use a definition of AONTs in terms of the entropy function *H*:

**Definition** **1.** *Let $X_{1},\cdots ,X_{s},Y_{1},\cdots ,Y_{s}$ be random variables taking values from the finite set X, with |X|=v. These 2s random variables define an (ℓ,s,v)-AONT provided that the following conditions, are satisfied:*

*1.*  *$H(Y_{1},\cdots ,Y_{s}|X_{1},\cdots ,X_{s})=0$,*
*2.*  $H(X_{1},\cdots ,X_{s}|Y_{1},\cdots ,Y_{s})=0$
*3.*  *For all $X\subseteq \{X_{1},\cdots ,X_{s}\}$ with |X|=ℓ, and for all $Y\subseteq \{Y_{1},\cdots ,Y_{s}\}$ with |Y|=ℓ, it holds that*
  (2) $$H(X|\left\{Y_{1},\cdots ,Y_{s}\right\}\backslash Y)=H\left(X\right).$$

The definition of a linear AONT is the obvious one:

**Definition** **2.** *Let X be a finite field. An all-or-nothing transform is* linear *if each $y_{i}$ is an X-linear function of $x_{1},\cdots ,x_{s}$.*

The following theorem provides a method to obtain linear AONTs [21]:

**Theorem** **1.** *Let q be a prime power and $M\in F_{q}^{s\times s}$ invertible. Then M defines a linear (ℓ,s,q)-AONT*

(3) $$\begin{array}{cccc} \phi : & {F_{q}}^{s} & ⟶ & {F_{q}}^{s}\\ \; & x & \mapsto & xM^{-1} \end{array}$$

*if and only if every ℓ by ℓ submatrix of M is invertible.*

## 3. Results

With this preparation, we are ready to discuss the type of encryption schemes we are interested in here more formally.

### 3.1. Symmetric-key Encryption Using Mesoscopic Coherent States

Motivated by AlphaEta, we consider symmetric key encryption, where parts of the ciphertext can be non-classical: it can be a sequence of coherent states. When decrypting a ciphertext, a measurement should be done to obtain a classical bitstring from the coherent states. This together with the classical part forms the *reconstructed ciphertext*. Afterwards, from the reconstructed ciphertext, the plaintext can be recovered:

**Definition** **3.** *A symmetric-key encryption scheme using mesoscopic coherent states is a triple of algorithms as follows:*

- KeyGen *: Given a key length, outputs a corresponding secret key k.*
- Enc *: Given a plaintext m and secret key k, it outputs a ciphertext c, consisting of a sequence of coherent states and a bitstring:*
  (4) $$c=(|\psi_{1}\rangle ,\cdots ,|\psi_{j}\rangle ,c_{1},\cdots ,c_{\ell })$$
- Dec *: This process consists of two phases. Given a ciphertext c and a secret key k, the sequence of coherent states in c is measured in the first phase. Now c can be considered a classical bitstring when entering the second phase of the decryption. The final output of the algorithm is the plaintext m.*

**Remark** **1.** *We allow the sequence of coherent states or the classical bitstring to be empty to include both classical and purely quantum symmetric-key encryption schemes, such as AlphaEta.*

**Figure 1:**
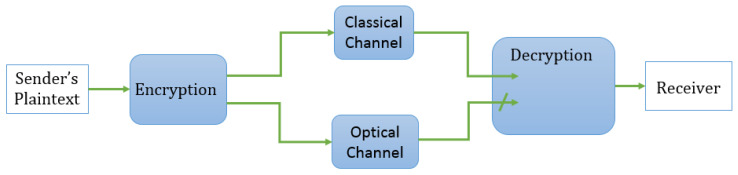
Overview of a symmetric-key encryption scheme using mesoscopic coherent states.

Figure 1 illustrates the overall structure of a symmetric-key encryption scheme using mesoscopic coherent states, highlighting the two different communication channels.

For such a scheme to be useful, we assume that the intended recipient, knowing the secret key and leaving aside channel imperfections, can recover correctly the plaintext from the ciphertext. This is captured by correctness:

**Definition** **4.** *A symmetric-key encryption scheme using mesoscopic coherent states is*δ-correct* if $\left({\mathrm{Dec}}_{k}\circ {\mathrm{Enc}}_{k}\right)\left(m\right)=m$ except with a probability smaller than δ, for all k generated by KeyGen and all plaintexts m.*

Notice that AlphaEta satisfies Definition 4, as discussed in [10]; the intended recipient can recover a plaintext bit with an error rate below $10^{-9}$.

A user or adversary not knowing the secret information, should obtain as little information about a plaintext bit as possible. The entropy of a plaintext bit given the reconstructed ciphertext should not be very different from the entropy about that plaintext bit. We use this as motivation for the following security definition. One could consider stronger security notions, in particular when allowing ciphertext expansion, but the following notion seems adequate when aiming at a composition with a classical scheme as discussed in Section 4.1.

**Definition** **5.** *Let (KeyGen,Enc,Dec) be a symmetric-key encryption scheme using mesoscopic coherent states. Let $P_{i}$ denote the random variable taking the value of a plaintext bit and $C_{i}$ denote the random variables taking the value of classical ciphertext bits. Let $\tilde{C_{1}},\cdots ,\tilde{C_{l}}$ represent the random variables taking the value of the reconstructed ciphertext bits, i.e, $\tilde{C_{i}}$s correspond to the bits obtained after the corresponding measurement of the coherent states. We say that (KeyGen,Enc,Dec) is*ϵ-secure* if for 1≤i≤s:*

(5) $$|H\left(P_{i}|\tilde{C_{1}},\cdots ,\tilde{C_{\ell }},C_{\ell +1},\cdots ,C_{s}\right)-H\left(P_{i}\right)|\leq \epsilon$$

**Remark** **2.** *The AlphaEta protocol satisfies Definition 5, assuming the a priori probability distribution for a bit is the discrete uniform probability.*

*As discussed in [10], the amount of information that the adversary, not knowing the bases, can obtain through eavesdropping can be made sufficiently small by choosing the parameters 〈n〉 and β appropriately. For example, for 〈n〉=100 and β=5, the minimum probability of error is $P_{e}^{E}\approx 0.476$. So $H\left(P|\tilde{C}\right)=-P_{e}^{E}\log_{2}P_{e}^{E}-\left(1-P_{e}^{E}\right)\log_{2}P_{e}^{E}\approx 0.998$. Since H(P)=1, this will imply AlphaEta is ϵ-secure for ϵ=0.002 for the chosen parameters.*

*Moreover, the mutual information of the plaintext bit and reconstructed ciphertext is equal to ϵ, since $I\left(P,\tilde{C}\right)=H\left(P\right)-H\left(P|\tilde{C}\right)$. A graph of $P_{e}^{E}$ and $I(P,\tilde{C})$ as a function of 〈n〉 and β can be found in [10], where it can be seen how they can be made as close to 1/2 and 0, respectively, as desired by choosing the appropriate parameters.*

Once the secret key is exposed, previous transmissions using the exposed key could become vulnerable. An adversary may have obtained previous ciphertexts, which, knowing the secret key, could potentially be decrypted and the messages would be known. Owing to the fact that in a symmetric-key encryption scheme using mesoscopic coherent states, parts of the ciphertext may be non-classical and we assume the adversary to conduct measurements on those parts, we can hope for some type of forward secrecy, however:

**Definition** **6.** *For t∈N, let $P_{i}^{t}$ be the random variable taking the value of a plaintext bit, and $C_{i}^{t}$ the random variables taking the value of classical ciphertext bits at time t. Let $\tilde{C_{1}^{t}},\cdots ,\tilde{C_{\ell }^{t}}$ represent the random variables taking the value of the reconstructed ciphertext bits, i.e., $\tilde{C_{i}^{t}}$s correspond to the bits obtained after the corresponding measurement of coherent states at time t. Let $K^{1},\cdots ,K^{t}$ represent the sequence of random variables taking the values of the symmetric key, i.e., values in ${\left\{0,\cdots ,\beta -1\right\}}^{s}$. We assume after time t (when the ciphertext has been sent and the measurements without knowing $K^{t}$ have been realized), $K^{1},\cdots ,K^{t}$ is revealed. A symmetric-key encryption scheme using mesoscopic coherent states is*ϵ-forward secure *if for all t we have*

(6) $$|H\left(P_{i}^{t}|\tilde{C_{1}^{t}},\cdots ,\tilde{C_{\ell }^{t}},C_{\ell +1}^{t},\cdots ,C_{s}^{t},K^{1},\cdots ,K^{t}\right)-H\left(P_{i}^{t}\right)|\leq \epsilon .$$

### 3.2. A Hybrid Construction

Instead of applying a symmetric-key encryption scheme using mesoscopic coherent states directly to plaintexts, we will apply preprocessing. This will enable us to send (large) parts of the ciphertexts over a (potentially cheaper) classical communication channel without sacrificing security. One benefit is that the somewhat subtle problem of error correction on the optical channel, can be localized to a smaller payload.

#### 3.2.1. Description and Design Rationale

Suppose party *A* wants to send an *s*-bit message to party *B*. We would like to invoke a linear AONT to transform the ciphertext such that if some blocks are missing, the entropy about other blocks is not reduced. We are particularly interested in the situation q=2 and l=1, with field elements representing bits. We try to “hide” individual input bits of an AONT where almost the complete output of the AONT is potentially available to an adversary. Unfortunately, the conditions of Theorem 1 cannot be satisfied when ℓ=1, q=2 and s≥2, since the matrix in which all entries equal 1 is not invertible. To fix this issue, consider an *s* by *s* matrix *M* with entries in $F_{2}$ with exactly s−1 entries equal to zero. From [21] (Lemma 7), *M* is invertible over $F_{2}$ if and only if the zero entries occur in s−1 different rows and in s−1 different columns. We will consider such matrices *M* with the zero-entries being arranged as follows:

$$M=\left[\begin{array}{cccccc} 1 & 1 & 1 & 1 & \cdots & 1\\ 1 & 0 & 1 & 1 & \cdots & 1\\ 1 & 1 & 0 & 1 & \cdots & 1\\ \vdots & \vdots & \vdots & \vdots & \ddots & \vdots \\ 1 & 1 & 1 & 1 & \cdots & 0 \end{array}\right]$$

So the first row of *M* contains all entries qual to 1. This means that each $x_{j}$ value of the input depends on the value $y_{1}$ of the output. Thus, if $y_{1}$ is unknown, any value of the input is completely undetermined. Therefore, if we consider this restriction, the above linear transformation *M* behaves as an AONT. As with Definition 1, we can define a restricted AONT.

**Definition** **7.** *Let $X_{1},\cdots ,X_{s},Y_{1},\cdots ,Y_{s}$ be random variables taking on values in the finite set X. These 2s random variables define a {1}-restricted AONT provided that the following conditions are satisfied:*

1. $H(Y_{1},\cdots ,Y_{s}|X_{1},\cdots ,X_{s})=0$
2. $H(X_{1},\cdots ,X_{s}|Y_{1},\cdots ,Y_{s})=0$
3. *For all i such that 1≤i≤s, $H\left(X_{i}|Y_{2},\cdots ,Y_{s}\right)=H\left(X_{i}\right)$.*

We can generalize Definition 7 to an Υ-restricted AONT.

**Definition** **8.** *Let $X_{1},\cdots ,X_{s},Y_{1},\cdots ,Y_{s}$ be random variables taking on values in the finite set X. These 2s random variables define an Υ-restricted AONT provided that the following conditions are satisfied:*

1. $H(Y_{1},\cdots ,Y_{s}|X_{1},\cdots ,X_{s})=0$
2. $H(X_{1},\cdots ,X_{s}|Y_{1},\cdots ,Y_{s})=0$
3. *Let $Y=\{Y_{\upsilon }:\upsilon \in \Upsilon \}$ represent the collection of hidden bits. For all i such that 1≤i≤s, it holds that*
  (7) $$H(X_{i}|\left\{Y_{1},\cdots ,Y_{s}\right\}\backslash Y)=H\left(X_{i}\right).$$

For convenience, we will simply speak of an *ℓ-restricted AONT* instead of an {1,⋯,ℓ}-restricted AONT.

**Proposition** **1.** *Let $M\in {\mathrm{GL}}_{s}\left(F_{2}\right)$. Let $x=(x_{1}\;x_{2}\cdots \;x_{s})$ and $y=(y_{1}\;y_{2}\cdots \;y_{s})$ where x=yM. If ℓ coordinates of y are unknown, then there are $2^{\ell }$ possible preimages x.*

**Proof.**  Suppose one hides *l* values of y. Since each bit has two possibilities, this leads to $2^{l}$ possible choices for y. Since *M* is a bijection, it implies that there are also $2^{\ell }$ candidates for the correct preimage x. □

**Remark** **3.** *While being strong, one should note that the guarantees of our restricted AONT are also limited: if available, two $y_{i}$-values can be combined to obtain the corresponding* sum *of $x_{i}$-values. Specifically, one can express y in terms of x as $y=xM^{-1}$ where*

$$M^{-1}=\left[\begin{array}{ccccc} 0 & 1 & 1 & \cdots & 1\\ 1 & 1 & 0 & \cdots & 0\\ 1 & 0 & 1 & \cdots & 0\\ \vdots & \vdots & \vdots & \ddots & \vdots \\ 1 & 0 & 0 & \cdots & 1 \end{array}\right]$$

*if s is even. If s is odd, the form of $M^{-1}$ is similar except that $m_{11}=1$. It can be observed that $y_{i}=x_{1}+x_{i}$, for all i=2,⋯,s. Thus, one may take the sum of $y_{p}$ and $y_{q}$ to obtain the sum of $x_{p}$ and $x_{q}$ (for p,q≠1):*

(8) $$\begin{array}{cc} y_{p}+y_{q} & =x_{1}+x_{p}+x_{1}+x_{q}\\ \; & =x_{p}+x_{q} \end{array}$$

Proposition 1 applies to linear restricted AONTs, and we will proceed by applying an *ℓ*-restricted AONT as in Definition 8. The bits represented by Y will be transmitted to party *B* through the optical channel with AlphaEta, while the rest can be sent through a public classical channel.

It is crucial that the bits sent through the optical channel are received correctly. Otherwise, because of the properties of the (restricted) AONT, no bit of the plaintext could be recovered. Thus we will apply an error-correcting code to the bits indexed by Y beforehand. One concern here is the impact of this added redundancy on security, and we will stick here to an embarrassingly trivial approach: For each bit in Y, we will repeat it *r* times with *r* being odd. Barbosa pointed out that in this case the system can be designed to a desired security level $P_{e}^{E}$, through the correct choice of 〈n〉 and β [8]. Thus, even with the *r*-repeated sequence, the adversary’s error probability can be made close to 1/2.

**Figure 2:**
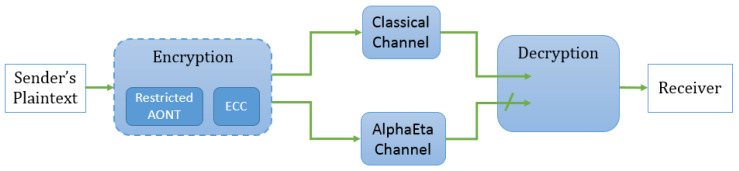
How a message is sent using the new construction.

**Figure 3:**
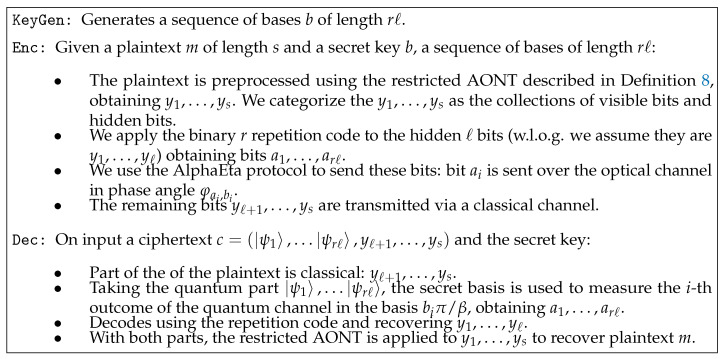
A quantum symmetric-key encryption scheme.

Figure 2 shows the main components of our protocol. A more detailed description of the algorithms as described in Definition 3 is provided in Figure 3.

**Remark** **4.** *The protocol describes how to encrypt a single s-bit plaintext block. To handle arbitrary length plaintexts, one could in a first approach break the plaintext into blocks of length s (using padding if needed) and apply the protocol block by block. However, more elaborate “modes of operation” could be explored and analyzed, e.g., a “tree construction": the restricted AONT is applied to multiple blocks individually and afterwards, several bits of the corresponding resulting blocks are chosen and fed into the restricted AONT again. After that step, one chooses the bits to be sent through the AlphaEta channel and the ones to be sent classically. We leave the analysis of such modes of operation to future work.*

#### 3.2.2. Security Analysis

The proposed hybrid construction, satisfies the notion of correctness and security as defined in Section 3.1:

**Proposition** **2.** *Let T be an ℓ-restricted AONT on $F_{2}^{s}$, let C be a binary repetition code of odd length r, and and assume that AlphaEta is δ-correct. Then the protocol in Figure 2 is $\delta^{\prime }$-correct according to Definition 4 where*

(9) δ′=1−∑i=0r−12riδi(1−δ)r−iℓ.

**Proof.**  The recipient receives all but rℓ elements of the plaintext through the classical channel and the missing rℓ elements via AlphaEta. Using the shared key of the AlphaEta protocol, since this protocol is δ-correct, the recipient recovers each of the corresponding rℓ elements with probability of error smaller than δ. Since C is a binary repetition code, each codeword containing up to (r−1)/2 errors can be decoded correctly. This can be done with probability greater than

(10) ∑i=0r−12riδi(1−δ)r−i.

Therefore, the recipient can recover all *ℓ* bits sent through the AlphaEta channel and therefore obtain the *s* elements of the plaintext with probability of error smaller than

(11) 1−∑i=0r−12riδi(1−δ)r−iℓ.

 □

**Remark** **5.** *By ([10] Theorem 1), for the AlphaEta protocol δ is less that $10^{-9}$. As shown in Section 4.2, this allows acceptably small values for $\delta^{\prime }$.*

In the proof of the following theorem, we are assuming the probability of $Y_{i}=0$ and $Y_{i}=1$ equals 1/2 to use the results in [8] and assume the measurements and the values sent through AlphaEta to be close to independent. In case we would like to provably ensure this as part of the protocol, we could apply a one-time pad (only) to the bits $a_{1},\cdots ,a_{r\ell }$ prior to sending them through the AlphaEta channel.

**Theorem** **2.** *Let T be an ℓ-restricted AONT on $F_{2}^{s}$, let C be a binary repetition code of odd length r, and assume that AlphaEta is ϵ-secure. Then the protocol in Figure 2 is ϵ-secure according to Definition 5.*

**Proof.**  We will use some properties about the entropy function. In particular, we recall the Chain Rule for entropy and a Corollary. For any random variables X,Y and *Z*, the following holds:

(12) $$\begin{array}{ccc} H(Y|X) & = & H(X,Y)-H(X) \end{array}$$

(13) $$\begin{array}{ccc} H(X|Z) & = & H(X,Y|Z)-H(Y|X,Z) \end{array}$$

Throughout the proof, we will use capital letters to denote the random variables taking the values of the corresponding plaintext and ciphertext bits.

The ciphertext for our protocol is of the form $c=(|\psi_{1}\rangle ,\cdots |\psi_{r\ell }\rangle ,y_{\ell +1},\cdots ,y_{s})$ as described in Figure 3 . Let $X_{i}$ denote the random variable corresponding to a bit of plaintext. After measuring the non-classical part, one should obtain $a_{1},\cdots ,a_{r\ell }$. Not knowing the bases, one obtains ${\tilde{a}}_{1},\cdots ,{\tilde{a}}_{r\ell }$. Thus, the reconstructed ciphertext is of the form $y={\tilde{a}}_{1},\cdots ,{\tilde{a}}_{r\ell },y_{\ell +1},\cdots ,y_{s}$. The entropy of the *i*-th bit of plaintext is hardly reduced after seeing the reconstructed ciphertext:

(14) $$\begin{array}{ccc} H(X_{i}|Y) & = & H(X_{i}|Y_{\ell +1},\cdots ,Y_{s},\tilde{A_{1}},\cdots ,\tilde{A_{r\ell }})\\ & = & H\left(Y_{\ell +1},\cdots ,Y_{s},X_{i}|\tilde{A_{1}},\cdots ,\tilde{A_{r\ell }}\right)-H\left(Y_{\ell +1},\cdots ,Y_{s}|\tilde{A_{1}},\cdots ,\tilde{A_{r\ell }}\right)\\ & = & H\left(Y_{\ell +1},\cdots ,Y_{s},X_{i}\right)-H\left(Y_{\ell +1},\cdots ,Y_{s}\right)-\epsilon \\ & = & H\left(X_{i}|Y_{\ell +1},\cdots ,Y_{s}\right)+H\left(Y_{\ell +1},\cdots ,Y_{s}\right)-H\left(Y_{\ell +1},\cdots ,Y_{s}\right)-\epsilon \\ & = & H(X_{i})-\epsilon \end{array}$$

The second equality comes from the Corollary of the chain rule of entropy. Since the AlphaEta protocol provides the guarantee that the $\tilde{A_{1}},\cdots ,\tilde{A_{r\ell }}$ are randomly independent from any $Y_{1},\cdots ,Y_{s}$ (as discussed in Section 2.1, seeing $\tilde{a_{i}}$ provides only a small information about the sent bits, ϵ), the third equality holds. By applying the Chain rule of entropy to the term $H(Y_{\ell +1},\cdots ,Y_{s},X_{i})$, we get the fourth equality. The last equality comes from the guarantee of our restricted AONT. □

### 3.3. Forward Security

**Figure 4:**
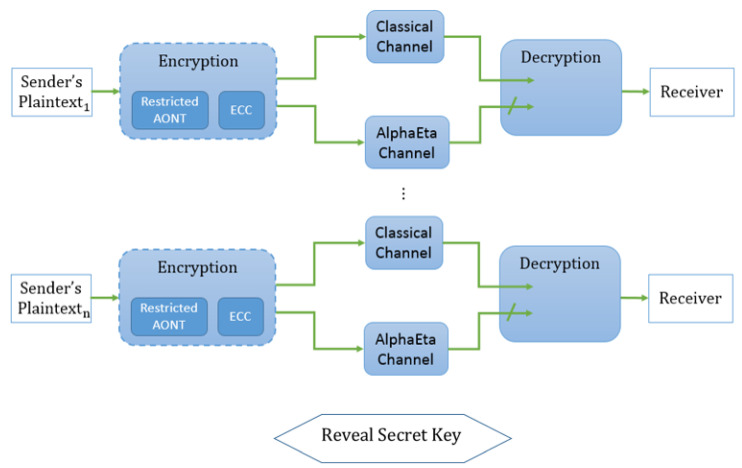
Description of an AlphaEta-based forward secure protocol.

Following the motivation in Section 3.1, we want to show that our hybrid construction satisfies the forward security property on a bit-wise level. We let $x_{i}^{t}$ represent a plaintext bit and $y^{t}=\tilde{a_{1}^{t}},\cdots ,\tilde{a_{rl}^{t}},y_{l+1}^{t},\cdots ,y_{s}^{t}$ represent the reconstructed ciphertext at time period *t* for t∈N. Let $k^{1},\cdots ,k^{t}$ represent the sequence of the secret keys. We assume after time *t* (when the ciphertext has been sent and the measurements without knowing $k^{t}$ have been realized), $k^{t}$ becomes public information. Even with knowledge of $k^{1},\cdots ,k^{t}$, the entropy of the random variable taking the value of a plaintext bit $X_{i}^{t}$ is hardly reduced:

**Theorem** **3.** *If AlphaEta is ϵ-secure in transmitting a single bit, C a binary repetition code with odd length r, and T an ℓ-restricted restricted AONT on $F_{2}^{s}$, then, the protocol in Figure 4 is ϵ-forward secure according to Definition 6.*

**Proof.**  According to Bayes’ Rule of entropy, for any random variables X,Y,

(15) H(Y|X)=H(X|Y)−H(X)+H(Y).

We will use capital letters to denote the random variables taking the values of the corresponding plaintext bits, reconstructed ciphertext and the keys. Hence, $H(X_{i}^{t}|Y^{t},K^{1},\cdots ,K^{t})$ is equal to

(16) $$\begin{array}{ccc} \; & H\left(Y^{t},X_{i}^{t}|K^{1},\cdots ,K^{t}\right)-H\left(Y^{t}|K^{1},\cdots ,K^{t}\right) & \;\left(\mathrm{Coroll}.\right)\\ = & H\left(K^{1},\cdots ,K^{t}|Y^{t},X_{i}^{t}\right)-H\left(K^{1},\cdots ,K^{t}\right)+\\ \; & H\left(Y^{t},X_{i}^{t}\right)-H\left(Y^{t}|K^{1},\cdots ,K^{t}\right) & \;(\mathrm{Bayes}’\;R.)\\ = & H\left(Y^{t},X_{i}^{t}\right)-H\left(Y^{t}|K^{1},\cdots ,K^{t}\right)\\ = & H\left(Y^{t}\right)+H\left(X_{i}^{t}|Y^{t}\right)-H\left(Y^{t}|K^{1},\cdots ,K^{t}\right) & \;(\mathrm{Chain}\;R.)\\ = & H\left(Y^{t}\right)+H\left(X_{i}^{t}\right)-\epsilon -H\left(Y^{t}|K^{1},\cdots ,K^{t}\right) & \;(\mathrm{Th}.2\;)\\ \geq & H\left(Y^{t}\right)+H\left(X_{i}^{t}\right)-H\left(Y^{t}\right)-\epsilon \\ = & H(X_{i}^{t})-\epsilon \end{array}$$

Since the secret keys $K^{1},\cdots ,K^{t}$ have been revealed, $H(K^{1},\cdots ,K^{t}|Y^{t},X_{i}^{t})$ as well as $H(K^{1},\cdots ,K^{t})$ are zero. Hence, the third equality holds. In addition, the inequality holds since $H\left(Y^{t}|K^{1},\cdots ,K^{t}\right)\leq H\left(Y^{t}\right)$. □

## 4. Discussion

In our security discussion of the protocol, the plaintext was assumed to be comprised of independently uniformly chosen bits. Moreover, we did so far not address the problem of ensuring authentication or integrity.

### 4.1. Integrating Classical Authenticated Encryption

A pragmatic approach to address these issues is to apply a (high-speed) authenticated encryption to the plaintext, prior to the use of the restricted AONT. Specifically, here we choose the encrypt-then-MAC approach, leveraging the popular combination of the ChaCha20 stream cipher and Poly1305 authenticator [22].

**Figure 5:**
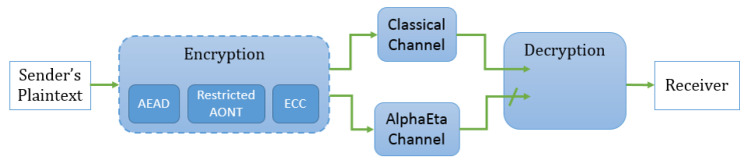
How the message is sent using the hybrid construction with authenticated encryption scheme; AEAD represents the application of ChaCha20 and Poly1305.

**Figure 6:**
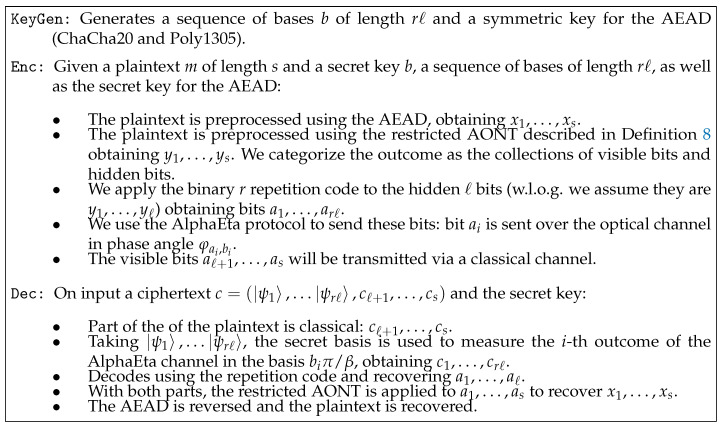
A symmetric-key encryption scheme using mesoscopic coherent states with incorporated AEAD.

Applying this combination results in a ciphertext that is (computationally) indistinguishable from random. (The ChaCha20 block function is a pseudo-random function (PRF) [23]. In addition, the last step of Poly1305 adds a fresh pseudo-random string, which can be derived using the ChaCha20 block function [22] and results in an authenticator that is (computationally) indistinguishable from random.) An overview of integrating such an additional classical preprocessing in our protocol is shown in Figure 5. Details are given in Figure 6.

### 4.2. Choosing Parameters

The inputs of the AEAD using ChaCha20 cipher and Poly1305 Authenticator include a 256-bit key, a 96-bit nonce, an arbitrary length plaintext, and an arbitrary length additional authenticated data. For simplicity, here we assume the latter to be empty, though one could consider a situation for our protocol where parts of the payload does not require confidentiality. The output of the AEAD is a ciphertext of the same length as the plaintext and a 128-bit tag. It seems reasonable to choose 128 bits as a block size for the input and output of the AEAD. For further detailed parameters of the AEAD, one can refer to [22].

Once we have preprocessed the plaintext using the AEAD, we will apply the restricted AONT. We use the linear construction based on the matrix $M^{-1}$ as defined in Section 3.2.1. To take advantage of the property of the restricted AONT, we would like its input to be greater than 128 bits. Let us consider the case where the inputs are 256-bit blocks. That means the matrix $M^{-1}$ will have dimension 256 by 256. The outputs of the restricted AONT are also 256-bit blocks. We collect the first 128 bits as the hidden bits to be sent over the AlphaEta channel. The remaining bits will be sent over the classical channel.

We apply a binary repetition code of odd length to our collection of hidden bits. Recall the probability of error for transmitting one bit:

(17) Perr=1−∑i=0r−12riδi(1−δ)r−i

where *r* is the length of the code and δ is the channel error probability. Table 1 (computed by means of the computer algebra system Magma [24]) demonstrates the probability of error $\delta^{\prime }$ for transmitting 128 hidden bits given the length of the binary repetition code and the channel error probability.

One can see the improvement in the correctness parameter of the symmetric-key encryption scheme using mesoscopic coherent states.

## 5. Conclusions

In this paper, we give a definition for a symmetric-key encryption scheme using mesoscopic states, including a security definition for such a scheme. We provide an example of such schemes using AlphaEta in combination with a variant of a classical AONT and error correction. Leveraging both an optical and a classical communication channel, we obtain an efficient construction with an interesting (information-theoretic) security guarantee. A forward security property is from a classical point of view quite remarkable: even after revealing the complete secret key, due to the underlying physical principle, the individual bits of the payload still remain hidden. In combination with a classical authenticated encryption, our design creates a situation where an adversary, based on physical principles, does not have even access to the classical output of a cipher, adding a conceptually interesting layer of security to a classical cipher, this being the main advantage over classical solutions.

When using repetition codes in our construction, the correctness parameter is improved, while the security parameter does not change, and only some part of the ciphertext is transmitted through the optical channel, which poses an advantage with respect to purely quantum schemes. From an implementation point of view, it deserves noting that our design integrates naturally with existing experimental setups for AlphaEta. All the steps we add can be seen as pre-processing and post-processing of the payload, cf. Figure 2. Therefore, for potential users, e.g., in industry, the experimentally demanding implementation of the optical channel does not have to be altered to be able to benefit from the security guarantees our approach offers. In addition, if one is willing to work with heuristic security arguments, the integration of classical authenticated encryption as outlined in Section 4.1 appears fairly attractive.

In general, symmetric-key encryption schemes using mesoscopic states as defined here, should interface naturally with schemes like AlphaEta, as the cryptographic processing assumes certain (abstract) guarantees provided by the invoked optical channel only. In this paper we leave the integration of more involved error correction techniques as future work; it seems fair to say that already our basic design offers a reasonably efficient combination of classical and physical techniques for securing a data transmission.

## Figures and Tables

**Table 1:** Probability of error for transmitting 128 bits with a repetition code.

*r*	$\frac{r-1}{2}$	δ	$\delta^{\prime }$
3	1	$10^{-9}$	$3.84\times 10^{-16}$
		$10^{-5}$	$3.84\times 10^{-8}$
		$10^{-1}$	0.9736
7	3	$10^{-9}$	$4.48\times 10^{-33}$
		$10^{-5}$	$4.48\times 10^{-17}$
		$10^{-1}$	0.2951
101	50	$10^{-9}$	$2.56\times 10^{-428}$
		$10^{-5}$	$2.56\times 10^{-224}$
		$10^{-1}$	$1.47\times 10^{-22}$
